# 2-[3-(2-Acetoxyphenyl)quinoxa­lin-2-yl]phenyl acetate

**DOI:** 10.1107/S1600536813009161

**Published:** 2013-04-20

**Authors:** Dan-Feng Shen, Shao-Jie Lou, Dan-Qian Xu

**Affiliations:** a State Key Laboratory Breeding Base of Green Chemistry-Synthesis Technology, Zhejiang University of Technology, Hangzhou, 310014, People’s Republic of China

## Abstract

The title compound, C_24_H_18_N_2_O_4_, crystallizes as a *syn*-conformer, with dihedral angles between the quinoxaline moiety and the acet­oxy-substituted benzene rings of 53.46 (3)° and 54.78 (3)°. In the crystal, the mol­ecules form chains along [100] *via* C—H⋯O inter­actions.

## Related literature

For general background to quinoxaline derivatives, see: Brasche & Buchwald (2008 ▶); Do & Daugulis (2008 ▶); He *et al.* (2003 ▶); Kim *et al.* (2004 ▶); Lyons & Sanford (2010 ▶). For quinoxaline-directed C—H activation, see: Reddy *et al.* (2011 ▶). For a related structure, see: Rajnikant *et al.* (2006 ▶).
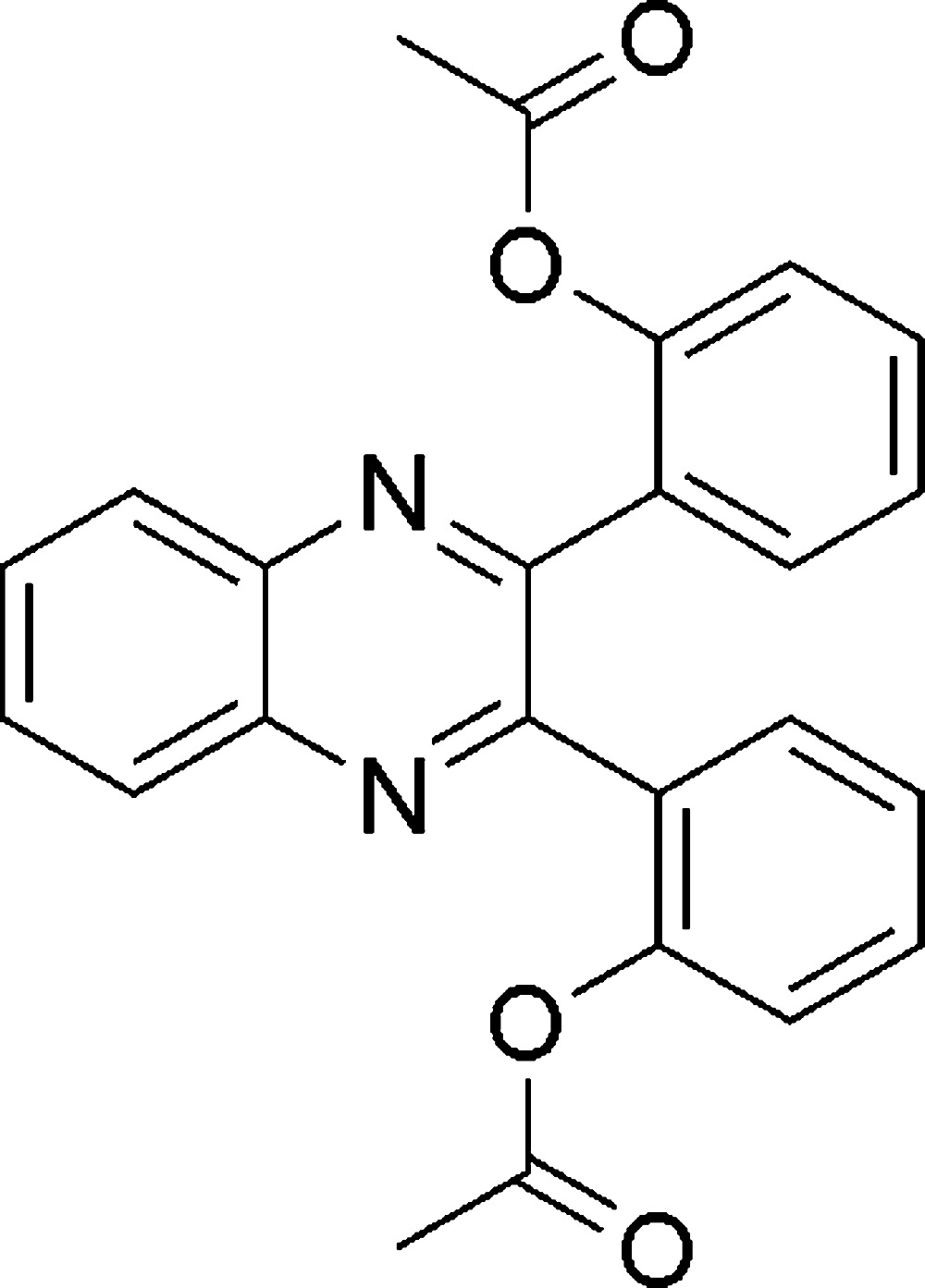


## Experimental

### 

#### Crystal data

C_24_H_18_N_2_O_4_
*M* *_r_* = 398.40
Monoclinic, 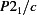

*a* = 9.5723 (5) Å
*b* = 16.7309 (8) Å
*c* = 13.0555 (6) Å
β = 92.929 (2)°
*V* = 2088.15 (18) Å^3^
*Z* = 4
Mo *K*α radiation
μ = 0.09 mm^−1^
*T* = 296 K
0.48 × 0.46 × 0.20 mm

#### Data collection

Rigaku R-AXIS RAPID/ZJUG diffractometer
Absorption correction: multi-scan (*ABSCOR*; Higashi, 1995 ▶) *T*_min_ = 0.959, *T*_max_ = 0.983
15632 measured reflections
3646 independent reflections
2553 reflections with *I* > 2σ(*I*)
*R*_int_ = 0.043

#### Refinement

*R*[*F* ^2^ > 2σ(*F* ^2^)] = 0.045
*wR*(*F* ^2^) = 0.117
*S* = 1.00
3646 reflections
274 parameters
H-atom parameters constrained
Δρ_max_ = 0.17 e Å^−3^
Δρ_min_ = −0.20 e Å^−3^

### 

Data collection: *PROCESS-AUTO* (Rigaku, 2006 ▶); cell refinement: *PROCESS-AUTO*; data reduction: *CrystalStructure* (Rigaku, 2007 ▶); program(s) used to solve structure: *SHELXS97* (Sheldrick, 2008 ▶); program(s) used to refine structure: *SHELXL97* (Sheldrick, 2008 ▶); molecular graphics: *ORTEP-3 for Windows* (Farrugia, 2012 ▶); software used to prepare material for publication: *WinGX* (Farrugia, 2012 ▶).

## Supplementary Material

Crystal structure: contains datablock(s) global, I. DOI: 10.1107/S1600536813009161/ld2099sup1.cif

Structure factors: contains datablock(s) I. DOI: 10.1107/S1600536813009161/ld2099Isup2.hkl

Supplementary material file. DOI: 10.1107/S1600536813009161/ld2099Isup3.cml

Additional supplementary materials:  crystallographic information; 3D view; checkCIF report

## Figures and Tables

**Table 1:** Hydrogen-bond geometry (Å, °)

*D*—H⋯*A*	*D*—H	H⋯*A*	*D*⋯*A*	*D*—H⋯*A*
C13—H13⋯O4^i^	0.93	2.43	3.329 (2)	164

Symmetry code: (i) 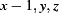.

**Fig. 1.**
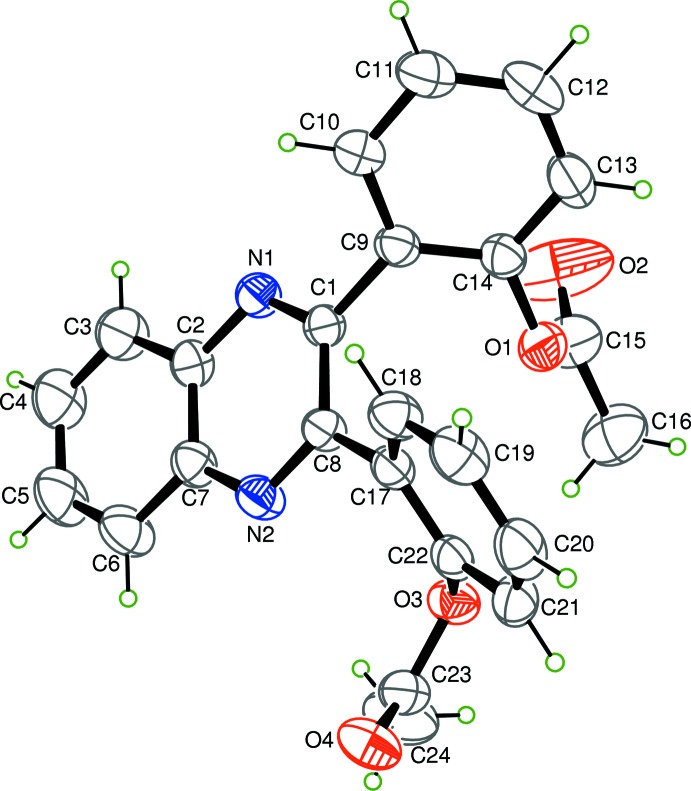
The molecule of the title compound, with the atomic labeling scheme. Displacement ellipsoids are drawn at the 40% probability level.

## supplementary crystallographic information

### Comment

Quinoxaline derivatives have received considerable interest from chemists because of their pharmacological properties such as antiviral, antibacterial, anti-inflammatory, and antiprotozoal activities, and as kinase inhibitors. C—H activation is a versatile approach for the direct functionalization of aromatic C—H bonds using transition metal catalysis. Palladium complexes are particularly attractive catalysts for such transformations. The molecular structure of the title compound is presented on Fig. 1. The plane of the quinoxaline moiety makes angles of 53.46 (3)°, 54.78 (3)° with the planes of phenyl rings. The torsion angle C9—C1—C8—C17 is 4.91 (3)°.

### Experimental

A mixture of 2,3-diphenylquinoxaline (282 mg,1.0 mmol), phenyliodine diacetate (805 mg, 2.5 mmol), and Palladium acetate (34 mg, 0.15 mmol) in acetic acid-acetic anhydride (3.0 ml-3.0 ml) was stirred at room temperature for 10 min, then the resulting mixture was heated to 120 degrees for 4hr. After completion of reaction as indicated by TLC, the reaction mixture was filtered, diluted with water and extracted with dichloromethane. The combined organic layers were dried over anhydrous sodium sulfate salt, concentrated *in vacuo*, and purified by column chromatography on silica gel (eluent:petroleum ether-ethyl acetate) to afford pure product with a 90 percent yield. Suitable crystals were obtained by slow evaporation of an ethanol solution.

### Refinement

H atoms were placed in calculated position with C—H ranging from 0.93 Å to 0.98 Å. All H atoms included in the final cycles of refinement as riding mode, with *U*_iso_(H)=1.2*U*_eq_ of the carrier atoms. Positions of hydrogens of the methyl groups awere optimized rotationally.

### Crystal data

**Table:**

C_24_H_18_N_2_O_4_	*F*(000) = 832
*M**_r_* = 398.40	*D*_x_ = 1.267 Mg m^−^^3^
Monoclinic, *P*2_1_/*c*	Mo *K*α radiation, λ = 0.71073 Å
Hall symbol: -P 2ybc	Cell parameters from 11299 reflections
*a* = 9.5723 (5) Å	θ = 3.1–27.4°
*b* = 16.7309 (8) Å	µ = 0.09 mm^−^^1^
*c* = 13.0555 (6) Å	*T* = 296 K
β = 92.929 (2)°	Platelet, yellow
*V* = 2088.15 (18) Å^3^	0.48 × 0.46 × 0.20 mm
*Z* = 4	

### Data collection

**Table:**

Rigaku R-AXIS RAPID/ZJUG diffractometer	3646 independent reflections
Radiation source: rotating anode	2553 reflections with *I* > 2σ(*I*)
Graphite monochromator	*R*_int_ = 0.043
Detector resolution: 10.00 pixels mm^-1^	θ_max_ = 25.0°, θ_min_ = 3.1°
ω scans	*h* = −11→11
Absorption correction: multi-scan (*ABSCOR*; Higashi, 1995)	*k* = −19→19
*T*_min_ = 0.959, *T*_max_ = 0.983	*l* = −15→15
15632 measured reflections	

### Refinement

**Table:**

Refinement on *F*^2^	Secondary atom site location: difference Fourier map
Least-squares matrix: full	Hydrogen site location: inferred from neighbouring sites
*R*[*F*^2^ > 2σ(*F*^2^)] = 0.045	H-atom parameters constrained
*wR*(*F*^2^) = 0.117	*w* = 1/[σ^2^(*F*_o_^2^) + (0.045*P*)^2^ + 0.7545*P*] where *P* = (*F*_o_^2^ + 2*F*_c_^2^)/3
*S* = 1.00	(Δ/σ)_max_ = 0.002
3646 reflections	Δρ_max_ = 0.17 e Å^−^^3^
274 parameters	Δρ_min_ = −0.20 e Å^−^^3^
0 restraints	Extinction correction: *SHELXL*, Fc^*^=kFc[1+0.001xFc^2^λ^3^/sin(2θ)]^-1/4^
Primary atom site location: structure-invariant direct methods	Extinction coefficient: 0.045 (2)

### Special details

**Table:**

Geometry. All e.s.d.'s (except the e.s.d. in the dihedral angle between two l.s. planes) are estimated using the full covariance matrix. The cell e.s.d.'s are taken into account individually in the estimation of e.s.d.'s in distances, angles and torsion angles; correlations between e.s.d.'s in cell parameters are only used when they are defined by crystal symmetry. An approximate (isotropic) treatment of cell e.s.d.'s is used for estimating e.s.d.'s involving l.s. planes.
Refinement. Refinement of *F*^2^ against ALL reflections. The weighted *R*-factor *wR* and goodness of fit *S* are based on *F*^2^, conventional *R*-factors *R* are based on *F*, with *F* set to zero for negative *F*^2^. The threshold expression of *F*^2^ > σ(*F*^2^) is used only for calculating *R*-factors(gt) *etc*. and is not relevant to the choice of reflections for refinement. *R*-factors based on *F*^2^ are statistically about twice as large as those based on *F*, and *R*- factors based on ALL data will be even larger.

### Fractional atomic coordinates and isotropic or equivalent isotropic displacement parameters (Å^2^)

**Table:**

	*x*	*y*	*z*	*U*_iso_*/*U*_eq_	
C1	0.57616 (19)	0.12138 (11)	0.27152 (13)	0.0401 (4)	
C2	0.7311 (2)	0.02174 (11)	0.31884 (15)	0.0467 (5)	
C3	0.7625 (3)	−0.04950 (13)	0.37296 (19)	0.0678 (6)	
H3	0.6940	−0.0747	0.4093	0.081*	
C4	0.8934 (3)	−0.08127 (15)	0.3718 (2)	0.0778 (7)	
H4	0.9141	−0.1280	0.4080	0.093*	
C5	0.9964 (3)	−0.04432 (15)	0.3169 (2)	0.0783 (7)	
H5	1.0850	−0.0671	0.3166	0.094*	
C6	0.9699 (2)	0.02435 (14)	0.2639 (2)	0.0704 (7)	
H6	1.0394	0.0480	0.2270	0.085*	
C7	0.8360 (2)	0.05939 (12)	0.26533 (15)	0.0494 (5)	
C8	0.68513 (19)	0.16210 (11)	0.22108 (14)	0.0429 (5)	
C9	0.43173 (19)	0.15473 (11)	0.27655 (14)	0.0438 (5)	
C10	0.3759 (2)	0.16739 (13)	0.37183 (17)	0.0586 (6)	
H10	0.4290	0.1545	0.4312	0.070*	
C11	0.2436 (3)	0.19857 (15)	0.3797 (2)	0.0714 (7)	
H11	0.2083	0.2068	0.4439	0.086*	
C12	0.1641 (2)	0.21744 (15)	0.2926 (2)	0.0731 (7)	
H12	0.0751	0.2389	0.2980	0.088*	
C13	0.2152 (2)	0.20474 (14)	0.19734 (19)	0.0628 (6)	
H13	0.1610	0.2171	0.1383	0.075*	
C14	0.3478 (2)	0.17335 (12)	0.19050 (15)	0.0473 (5)	
C15	0.3570 (3)	0.09580 (15)	0.04113 (19)	0.0727 (7)	
C16	0.4121 (3)	0.09362 (19)	−0.0632 (2)	0.0940 (9)	
H16A	0.3871	0.0438	−0.0956	0.141*	
H16B	0.5121	0.0987	−0.0581	0.141*	
H16C	0.3727	0.1369	−0.1033	0.141*	
C17	0.66704 (19)	0.24376 (11)	0.17661 (15)	0.0432 (5)	
C18	0.6188 (2)	0.30646 (12)	0.23494 (17)	0.0537 (5)	
H18	0.5930	0.2967	0.3015	0.064*	
C19	0.6084 (2)	0.38308 (13)	0.19573 (19)	0.0622 (6)	
H19	0.5755	0.4244	0.2357	0.075*	
C20	0.6466 (2)	0.39806 (14)	0.09761 (19)	0.0650 (6)	
H20	0.6390	0.4495	0.0710	0.078*	
C21	0.6961 (2)	0.33717 (13)	0.03828 (17)	0.0577 (6)	
H21	0.7231	0.3475	−0.0279	0.069*	
C22	0.70544 (19)	0.26088 (11)	0.07788 (15)	0.0448 (5)	
C23	0.8838 (2)	0.19247 (13)	−0.00467 (16)	0.0554 (5)	
C24	0.9139 (3)	0.11638 (17)	−0.0569 (2)	0.0940 (9)	
H24A	1.0092	0.1164	−0.0765	0.141*	
H24B	0.8524	0.1108	−0.1169	0.141*	
H24C	0.8997	0.0726	−0.0111	0.141*	
N1	0.59936 (17)	0.05326 (9)	0.32027 (12)	0.0467 (4)	
N2	0.81105 (17)	0.13064 (10)	0.21658 (13)	0.0513 (4)	
O1	0.40006 (15)	0.16132 (9)	0.09276 (10)	0.0569 (4)	
O2	0.2849 (3)	0.04774 (14)	0.0780 (2)	0.1502 (12)	
O3	0.74576 (14)	0.19711 (8)	0.01622 (10)	0.0536 (4)	
O4	0.96565 (16)	0.24378 (10)	0.01729 (13)	0.0718 (5)	

### Atomic displacement parameters (Å^2^)

**Table:**

	*U*^11^	*U*^22^	*U*^33^	*U*^12^	*U*^13^	*U*^23^
C1	0.0399 (11)	0.0407 (10)	0.0397 (9)	−0.0011 (8)	0.0016 (8)	−0.0007 (8)
C2	0.0485 (12)	0.0394 (11)	0.0519 (11)	0.0026 (9)	−0.0016 (9)	0.0003 (8)
C3	0.0670 (16)	0.0531 (13)	0.0834 (16)	0.0067 (11)	0.0054 (12)	0.0188 (12)
C4	0.0777 (19)	0.0577 (15)	0.0972 (19)	0.0184 (13)	−0.0026 (15)	0.0172 (13)
C5	0.0593 (16)	0.0675 (16)	0.108 (2)	0.0249 (13)	−0.0009 (14)	0.0111 (14)
C6	0.0494 (14)	0.0646 (15)	0.0979 (18)	0.0147 (11)	0.0098 (12)	0.0115 (13)
C7	0.0457 (12)	0.0445 (11)	0.0580 (12)	0.0051 (9)	0.0015 (9)	0.0023 (9)
C8	0.0384 (11)	0.0427 (11)	0.0477 (11)	0.0002 (8)	0.0036 (8)	0.0007 (8)
C9	0.0381 (11)	0.0445 (11)	0.0492 (11)	−0.0024 (8)	0.0060 (9)	0.0029 (8)
C10	0.0454 (13)	0.0746 (15)	0.0564 (12)	0.0038 (11)	0.0086 (10)	0.0019 (10)
C11	0.0522 (15)	0.0910 (18)	0.0725 (16)	0.0059 (13)	0.0183 (12)	−0.0046 (13)
C12	0.0405 (13)	0.0788 (17)	0.101 (2)	0.0113 (11)	0.0109 (13)	−0.0055 (14)
C13	0.0432 (13)	0.0661 (15)	0.0781 (16)	0.0040 (10)	−0.0068 (11)	0.0070 (11)
C14	0.0409 (12)	0.0490 (12)	0.0520 (11)	−0.0037 (9)	0.0017 (9)	0.0033 (9)
C15	0.0844 (18)	0.0636 (15)	0.0710 (16)	−0.0143 (14)	0.0131 (14)	−0.0085 (12)
C16	0.115 (2)	0.103 (2)	0.0653 (16)	−0.0082 (18)	0.0090 (16)	−0.0149 (15)
C17	0.0332 (10)	0.0432 (11)	0.0529 (11)	−0.0009 (8)	0.0005 (8)	0.0054 (8)
C18	0.0493 (13)	0.0485 (12)	0.0640 (13)	0.0034 (9)	0.0088 (10)	0.0036 (10)
C19	0.0546 (14)	0.0455 (13)	0.0865 (17)	0.0056 (10)	0.0044 (12)	0.0014 (11)
C20	0.0543 (14)	0.0490 (13)	0.0905 (17)	0.0007 (11)	−0.0080 (12)	0.0190 (12)
C21	0.0513 (13)	0.0609 (14)	0.0600 (13)	−0.0052 (10)	−0.0063 (10)	0.0185 (11)
C22	0.0328 (10)	0.0477 (11)	0.0531 (11)	−0.0055 (8)	−0.0048 (8)	0.0040 (9)
C23	0.0490 (13)	0.0624 (14)	0.0552 (12)	−0.0057 (11)	0.0080 (10)	0.0018 (10)
C24	0.0787 (19)	0.088 (2)	0.118 (2)	−0.0099 (15)	0.0290 (17)	−0.0343 (17)
N1	0.0458 (10)	0.0428 (9)	0.0517 (9)	−0.0007 (7)	0.0033 (7)	0.0048 (7)
N2	0.0399 (10)	0.0489 (10)	0.0656 (11)	0.0035 (7)	0.0075 (8)	0.0065 (8)
O1	0.0542 (9)	0.0661 (10)	0.0501 (8)	−0.0107 (7)	−0.0014 (7)	0.0030 (7)
O2	0.218 (3)	0.0990 (16)	0.142 (2)	−0.0865 (18)	0.092 (2)	−0.0508 (14)
O3	0.0461 (8)	0.0589 (9)	0.0562 (8)	−0.0123 (6)	0.0052 (6)	−0.0055 (6)
O4	0.0485 (10)	0.0730 (11)	0.0944 (12)	−0.0164 (8)	0.0085 (8)	−0.0079 (9)

### Geometric parameters (Å, º)

**Table:**

C1—N1	1.319 (2)	C13—H13	0.9300
C1—C8	1.434 (2)	C14—O1	1.409 (2)
C1—C9	1.495 (3)	C15—O2	1.178 (3)
C2—N1	1.368 (2)	C15—O1	1.341 (3)
C2—C7	1.401 (3)	C15—C16	1.486 (3)
C2—C3	1.410 (3)	C16—H16A	0.9600
C3—C4	1.362 (3)	C16—H16B	0.9600
C3—H3	0.9300	C16—H16C	0.9600
C4—C5	1.393 (4)	C17—C22	1.388 (3)
C4—H4	0.9300	C17—C18	1.389 (3)
C5—C6	1.359 (3)	C18—C19	1.382 (3)
C5—H5	0.9300	C18—H18	0.9300
C6—C7	1.410 (3)	C19—C20	1.373 (3)
C6—H6	0.9300	C19—H19	0.9300
C7—N2	1.367 (2)	C20—C21	1.379 (3)
C8—N2	1.319 (2)	C20—H20	0.9300
C8—C17	1.491 (3)	C21—C22	1.378 (3)
C9—C14	1.383 (3)	C21—H21	0.9300
C9—C10	1.395 (3)	C22—O3	1.403 (2)
C10—C11	1.378 (3)	C23—O4	1.188 (2)
C10—H10	0.9300	C23—O3	1.365 (2)
C11—C12	1.372 (4)	C23—C24	1.479 (3)
C11—H11	0.9300	C24—H24A	0.9600
C12—C13	1.376 (3)	C24—H24B	0.9600
C12—H12	0.9300	C24—H24C	0.9600
C13—C14	1.381 (3)		
			
N1—C1—C8	121.58 (17)	C9—C14—O1	119.04 (17)
N1—C1—C9	115.76 (16)	O2—C15—O1	121.6 (2)
C8—C1—C9	122.60 (16)	O2—C15—C16	126.9 (3)
N1—C2—C7	121.18 (17)	O1—C15—C16	111.5 (2)
N1—C2—C3	119.48 (18)	C15—C16—H16A	109.5
C7—C2—C3	119.34 (19)	C15—C16—H16B	109.5
C4—C3—C2	119.8 (2)	H16A—C16—H16B	109.5
C4—C3—H3	120.1	C15—C16—H16C	109.5
C2—C3—H3	120.1	H16A—C16—H16C	109.5
C3—C4—C5	120.6 (2)	H16B—C16—H16C	109.5
C3—C4—H4	119.7	C22—C17—C18	117.67 (18)
C5—C4—H4	119.7	C22—C17—C8	121.31 (17)
C6—C5—C4	121.2 (2)	C18—C17—C8	120.91 (17)
C6—C5—H5	119.4	C19—C18—C17	121.1 (2)
C4—C5—H5	119.4	C19—C18—H18	119.4
C5—C6—C7	119.4 (2)	C17—C18—H18	119.4
C5—C6—H6	120.3	C20—C19—C18	119.8 (2)
C7—C6—H6	120.3	C20—C19—H19	120.1
N2—C7—C2	120.79 (17)	C18—C19—H19	120.1
N2—C7—C6	119.56 (19)	C19—C20—C21	120.3 (2)
C2—C7—C6	119.63 (19)	C19—C20—H20	119.8
N2—C8—C1	121.24 (17)	C21—C20—H20	119.8
N2—C8—C17	115.87 (16)	C22—C21—C20	119.4 (2)
C1—C8—C17	122.78 (16)	C22—C21—H21	120.3
C14—C9—C10	117.21 (18)	C20—C21—H21	120.3
C14—C9—C1	123.25 (17)	C21—C22—C17	121.62 (19)
C10—C9—C1	119.53 (17)	C21—C22—O3	120.24 (18)
C11—C10—C9	121.3 (2)	C17—C22—O3	118.01 (16)
C11—C10—H10	119.4	O4—C23—O3	123.0 (2)
C9—C10—H10	119.4	O4—C23—C24	126.2 (2)
C12—C11—C10	119.9 (2)	O3—C23—C24	110.8 (2)
C12—C11—H11	120.0	C23—C24—H24A	109.5
C10—C11—H11	120.0	C23—C24—H24B	109.5
C11—C12—C13	120.3 (2)	H24A—C24—H24B	109.5
C11—C12—H12	119.8	C23—C24—H24C	109.5
C13—C12—H12	119.8	H24A—C24—H24C	109.5
C12—C13—C14	119.2 (2)	H24B—C24—H24C	109.5
C12—C13—H13	120.4	C1—N1—C2	117.34 (16)
C14—C13—H13	120.4	C8—N2—C7	117.73 (16)
C13—C14—C9	122.07 (19)	C15—O1—C14	117.26 (16)
C13—C14—O1	118.89 (18)	C23—O3—C22	117.10 (15)
			
N1—C2—C3—C4	179.9 (2)	C1—C8—C17—C22	−131.55 (19)
C7—C2—C3—C4	−0.7 (3)	N2—C8—C17—C18	−124.0 (2)
C2—C3—C4—C5	−0.5 (4)	C1—C8—C17—C18	52.3 (3)
C3—C4—C5—C6	0.5 (4)	C22—C17—C18—C19	0.5 (3)
C4—C5—C6—C7	0.7 (4)	C8—C17—C18—C19	176.78 (19)
N1—C2—C7—N2	2.8 (3)	C17—C18—C19—C20	−0.2 (3)
C3—C2—C7—N2	−176.6 (2)	C18—C19—C20—C21	−0.4 (3)
N1—C2—C7—C6	−178.78 (19)	C19—C20—C21—C22	0.8 (3)
C3—C2—C7—C6	1.8 (3)	C20—C21—C22—C17	−0.5 (3)
C5—C6—C7—N2	176.6 (2)	C20—C21—C22—O3	175.29 (18)
C5—C6—C7—C2	−1.9 (4)	C18—C17—C22—C21	−0.1 (3)
N1—C1—C8—N2	3.9 (3)	C8—C17—C22—C21	−176.41 (18)
C9—C1—C8—N2	−178.99 (17)	C18—C17—C22—O3	−176.01 (17)
N1—C1—C8—C17	−172.21 (17)	C8—C17—C22—O3	7.7 (3)
C9—C1—C8—C17	4.9 (3)	C8—C1—N1—C2	−1.5 (3)
N1—C1—C9—C14	−124.4 (2)	C9—C1—N1—C2	−178.85 (16)
C8—C1—C9—C14	58.4 (3)	C7—C2—N1—C1	−1.7 (3)
N1—C1—C9—C10	54.9 (2)	C3—C2—N1—C1	177.72 (19)
C8—C1—C9—C10	−122.4 (2)	C1—C8—N2—C7	−2.7 (3)
C14—C9—C10—C11	−1.2 (3)	C17—C8—N2—C7	173.69 (17)
C1—C9—C10—C11	179.5 (2)	C2—C7—N2—C8	−0.5 (3)
C9—C10—C11—C12	0.3 (4)	C6—C7—N2—C8	−178.93 (19)
C10—C11—C12—C13	0.6 (4)	O2—C15—O1—C14	−4.3 (4)
C11—C12—C13—C14	−0.5 (4)	C16—C15—O1—C14	176.0 (2)
C12—C13—C14—C9	−0.5 (3)	C13—C14—O1—C15	−79.8 (3)
C12—C13—C14—O1	−179.3 (2)	C9—C14—O1—C15	101.3 (2)
C10—C9—C14—C13	1.3 (3)	O4—C23—O3—C22	−8.4 (3)
C1—C9—C14—C13	−179.44 (19)	C24—C23—O3—C22	171.6 (2)
C10—C9—C14—O1	−179.88 (17)	C21—C22—O3—C23	74.4 (2)
C1—C9—C14—O1	−0.6 (3)	C17—C22—O3—C23	−109.70 (19)
N2—C8—C17—C22	52.2 (3)		

### Hydrogen-bond geometry (Å, º)

**Table:**

*D*—H···*A*	*D*—H	H···*A*	*D*···*A*	*D*—H···*A*
C13—H13···O4^i^	0.93	2.43	3.329 (2)	164

Symmetry code: (i) *x*−1, *y*, *z*.

## References

[bb1] Brasche, G. & Buchwald, S. L. (2008). *Angew. Chem. Int. Ed.* **47**, 1932–1934. 10.1002/anie.200705420 18228236

[bb2] Do, H.-Q. & Daugulis, O. (2008). *J. Am. Chem. Soc.* **130**, 1128–1129. 10.1021/ja077862l PMC2536496 18181627

[bb3] Farrugia, L. J. (2012). *J. Appl. Cryst.* **45**, 849–854.

[bb4] He, W., Meyers, M. R., Hanney, B., Sapada, A., Blider, G., Galzeinski, H., Amin, D., Needle, S., Page, K., Jayyosi, Z. & Perrone, H. (2003). *Bioorg. Med. Chem. Lett.* **13**, 3097–3100. 10.1016/s0960-894x(03)00655-3 12941342

[bb5] Higashi, T. (1995). *ABSCOR* Rigaku Corporation, Tokyo, Japan.

[bb6] Kim, Y. B., Kim, Y. H., Park, J. Y. & Kim, S. K. (2004). *Bioorg. Med. Chem. Lett.* **14**, 541–544. 10.1016/j.bmcl.2003.09.086 14698199

[bb7] Lyons, T. W. & Sanford, M. S. (2010). *Chem. Rev.* **110**, 1147–1169. 10.1021/cr900184e PMC2836499 20078038

[bb8] Rajnikant, Dinesh,, Deshmukh, M. B., Jadhav, S. & Kanwal, P. (2006). *Acta Cryst.* E**62**, o2356–o2357.

[bb9] Reddy, B. V. S., Ramesh, K. & Yadav, J. S. (2011). *Synlett*, **2**, 169–172.

[bb10] Rigaku (2006). *PROCESS-AUTO* Rigaku Corporation, Tokyo, Japan.

[bb11] Rigaku (2007). *CrystalStructure* Rigaku Americas Corporation, The Woodlands, Texas, USA.

[bb12] Sheldrick, G. M. (2008). *Acta Cryst.* A**64**, 112–122. 10.1107/S0108767307043930 18156677

